# Role of chromatin assembly factor-1/p60 and poly [ADP-ribose] polymerase 1 in mycosis fungoides

**DOI:** 10.1007/s00428-020-02952-z

**Published:** 2020-10-24

**Authors:** Massimo Mascolo, Antonio Travaglino, Silvia Varricchio, Daniela Russo, Elena Sabattini, Claudio Agostinelli, Clara Bertuzzi, Antonello Baldo, Alessandro Pileri, Marco Picardi, Fabrizio Pane, Stefania Staibano

**Affiliations:** 1 Department of Advanced Biomedical Sciences, Pathology Section, School of Medicine, University of Naples “Federico II”, Via Sergio Pansini, 5, 80131 Naples, Italy; 2 Haematopathology Unit, Department of Experimental Diagnostic and Specialty Medicine, S. Orsola-Malpighi Hospital, University of Bologna, Bologna, Italy; 3 Department of Clinical Medicine and Surgery, Dermatology Section, University of Naples “Federico II”, Naples, Italy; 4 Dermatology Unit, Department of Experimental, Diagnostic and Specialty Medicine, University of Bologna, Bologna, Italy; 5 Department of Clinical Medicine and Surgery, Hematology Section, University of Naples “Federico II”, Naples, Italy

**Keywords:** Mycosis fungoides, Cutaneous lymphoma, Poly [ADP-ribose] polymerase 1, Chromatin assembly factor-1, Prognosis

## Abstract

Mycosis fungoides (MF) represents the most common type of cutaneous lymphoma. In the majority of patients, the disease has a slow evolution and a protracted course; however, a subset of patients shows poor oncologic outcomes. Unfortunately, there are no reliable prognostic markers for MF, and the currently available treatments are only effective in a minority of patients. This study aimed to evaluate the expression and clinical significance of PARP-1 and CAF-1/p60 in MF. Sixty-four MF representatives of the different stages of disease were assessed by immunohistochemistry for PARP-1 and CAF-1/p60. The association of PARP-1 and CAF-1/p60 with the MF stage and outcome was assessed by using Fisher’s exact test and Kaplan-Meier survival analysis with the Log-rank test; a *p* value < 0.05 was considered significant. PARP-1 was overexpressed in 57.9% of MF and was significantly associated with a MF stage > II (*p* = 0.034) but not with the risk of death (*p* = 0.237). CAF-1/p60 was overexpressed in 26.8% of MF and was significantly associated with decreased overall survival (*p* < 0.001) but not with the MF stage (*p* = 1). A significant association was found between PARP-1 overexpression and CAF-1/p60 overexpression (*p* = 0.0025). Simultaneous overexpression of PARP-1 and CAF-1/p60 was significantly associated with decreased overall survival (*p* < 0.001), although less strongly than CAF-1/p60 alone (*χ*^2^ = 14.916 vs 21.729, respectively). In MF, PARP-1 is overexpressed in advanced stages, while CAF-1/p60 is overexpressed in the cases with shorter overall survival, appearing as a significant prognostic marker. A role for PARP-1 inhibitors and anti-CAF-1/p60 targeted therapy may be reasonably hypothesized in MF.

**Electronic supplementary material:**

The online version of this article (10.1007/s00428-020-02952-z) contains supplementary material, which is available to authorized users.

## Introduction

Mycosis fungoides (MF) is the most common type of primary cutaneous lymphoma, accounting for about 50% of cases [1, 2]. The incidence of MF is nearly 5.6 person per million, with marked regional variations and a higher incidence in the Black population, in adults and elderly patients, and in males [3–6].

Clinically, MF is a multistep process moving from patches to more infiltrated plaques and, eventually, developing into tumors, with slow progression over the years or decades; less common clinical variants are also described [7, 8]. At advanced stages (IIB–IVB), the 10-year survival decreases to less than 40% [9, 10]. Few MF cases may undergo large cell transformation, characterized by an aggressive clinical course and a median survival of less than 2 years [11].

Treatment is often multidisciplinary, as it combines skin-directed and systemic therapies [12]. However, targeted therapies achieve a complete response in less than half of patients, while conventional chemotherapy is associated with short-lived responses and worse overall outcomes [13–15]. The only potential cure for MF is traditional non-myeloablative allogeneic stem cell transplantation, which an overall survival rate of 46% at 5 years [15].

Patients with an advanced stage of the disease are associated with poor prognosis with a median survival < 4 years, but of only 13 months in the presence of nodal involvement [16–20].

Although several prognostic markers have been studied in MF to identify early-stage cases at high risk of disease progression [21–24], to date none has been validated and integrated in the management of MF patients.

Recently, Lemchak et al. identified poly [ADP-ribose] polymerase 1 (PARP-1) as a possible prognostic biomarker in MF [25]. PARP-1 is a protein involved in DNA repair which has been used as a therapeutic target in several human cancers [26–28]. In the case of MF, patients with PARP-1 overexpression in the early phase of the disease subsequently progressed to advanced stages [25].

CAF-1 is a heterotrimeric protein complex formed of three subunits (p48, p60, and p150) [29–34]. The CAF-1/p60 subunit plays a crucial function in cell replication, and its deregulation is involved in several human solid malignancies [35–41]. Interestingly, CAF-1/p60 expression has been shown to have a prognostic significance in several human malignant tumors [42–49], appearing as a possible “multi-tumoral” prognostic marker.

We previously showed that a simultaneous overexpression of PARP-1 and CAF-1/p60 was a specific hallmark of aggressiveness in oral squamous cell carcinoma [46].

Until now, in the literature, no data are available about the expression of CAF-1/p60 in patients with MF, and, to our knowledge, the only study evaluating the expression of PARP-1 in MF evaluated only 19 cases [25]. On this account, we aimed to evaluate the prognostic significance of PARP-1 and CAF1/p60 in a selected series of MF at different stages of the disease.

## Materials and methods

### Study population

Formalin-fixed, paraffin-embedded (FFPE) tissue blocks of 64 MF biopsies (20 incisional biopsies and 44 punch biopsies), diagnosed from January 1994 to December 2019 and representative of different stages of the disease, were retrieved from the archives of the Department of Advanced Biomedical Sciences, Pathology Unit, University Federico II of Naples and Haematopathology Unit, Department of Experimental Diagnostic and Specialty Medicine, S. Orsola-Malpighi Hospital, University of Bologna. An expert panel of dermatologists (AB and AP) and pathologists (MM, DR, ES, and CA) confirmed the diagnosis according to the criteria of the WHO-EORTC Classification of Cutaneous Lymphoma.

Follow-up data were retrospectively retrieved from clinical records and pathological reports. Overall survival (OS) was established from diagnosis to death or to the last contact date. Follow-up data included complete skin examination and laboratory tests for all patients; computed tomography scan of the chest, abdomen, and pelvis and bone marrow biopsy were performed on follow-up only if they were positive at the time of diagnosis.

The study was performed according to the guidelines of the Institutional Ethics Committee, which, in agreement with the Italian law, with reference to the topics of the current research, do not ask for the Ethical Committee approval, and, according to the Declaration of Helsinki, requires, for studies based only on retrospective analyses on routine archival FFPE tissue, a written informed consent from the living patient, following the indication of Italian DLgs no. 196/03 (Codex on Privacy) at the time of surgery.

### Immunohistochemistry

Immunohistochemistry was performed as previously described [46]. Slides were evaluated blindly by four observers (MM, DR, ES, and CA) and the cases with discordance were discussed and resolved by consensus. In all cases, five fields (940 magnifications) were assessed, evaluating a minimum of 20 cells in the representative areas. The expression of PARP-1 and CAF-1/p60 was quantified according to the percentage of stained nuclei using a 0–100% scale. The expression of PARP-1 and CAF-1/p60 was assessed to the nearest 10% to define the most significant threshold for the analysis. Since the most significant results were found for the 50% threshold, positivity in ≥ 50% of cells was labeled as “overexpression.”

### Statistical analysis

Statistical associations among PARP-1 expression, CAF-1/p60 expression, and MF stage were assessed by using Fisher’s exact test; a *p* value < 0.05 was considered significant. Kaplan-Meier survival analysis and log-rank test with *χ*^2^ calculation were used to assess the impact of PARP-1 and CAF-1/p60 expression on the OS, with a significant *p* value < 0.05; Kaplan-Meier curves were used to report the results graphically. Statistical analyses were performed by using Statistical Package for Social Science (SPSS) 18.0 package (SPSS Inc., Chicago, IL, USA).

## Results

### Clinical and pathological parameters

The clinical data and pathological features of the patients and MF are listed in Table 1. The study population was composed of 64 patients, out of which 43 males (67.2%) and 21 females (32.8%), with a mean age of 59.4 years (range 29–96 years).

**Table 1:** Characteristics of the patients

Age (*n* = 64)	
Mean (range)	59.4 (29–96)
Sex (*n* = 64)	
Male	43 (67.2%)
Female	21 (32.8%)
Stage (*n* = 53)	
I	20 (37.7%)
• IA	9 (17%)
• IB	11 (20.8%)
II	20 (37.7%)
• IIA	7 (13.2%)
• IIB	13 (24.5%)
III	1 (1.9%)
• IIIA	1 (1.9%)
IV	12 (22.6%)
• IVA1	7 (13.2%)
• IVA2	5 (9.4%)
PARP-1 (*n* = 57)	
Not overexpressed	24 (42.1%)
• 0–9%	5 (8.8%)
• 10–19%	1 (1.8%)
• 20–29%	4 (7%)
• 30–39%	13 (22.8%)
• 40–49%	1 (1.8%)
Overexpressed	33 (57.9%)
• 50–59%	10 (17.5%)
• 60–69%	6 (10.5%)
• 70–79%	11 (19.3%)
• 80–89%	5 (8.8%)
• 90–100%	1 (1.8%)
CAF-1/p60 (*n* = 56)	
Not overexpressed	41 (73.2%)
• 0–9%	11 (19.5%)
• 10–19%	5 (8.9%)
• 20–29%	16 (28.6%)
• 30–39%	8 (14.3%)
• 40–49%	1 (1.8%)
Overexpressed	15 (26.8%)
• 50–59%	1 (1.8%)
• 60–69%	4 (7.1%)
• 70–79%	7 (12.5%)
• 80–89%	2 (3.6%)
• 90–100%	1 (1.8%)
Follow-up (*n* = 63)	
Mean duration (range), months	52.9 (12–180)
Dead	8 (12.7%)
Alive	55 (87.3%)
• With disease	28 (44.4%)
• With remission	15 (23.8%)
• NOS	12 (19%)

Information about MF stage was available for 53 patients (82.8%). MF stage was IA in 9 patients (17%), IB in 11 patients (20.8%), IIA in 7 patients (13.2%), IIB in 13 patients (24.5%), IIIA in 1 patient (1.9%), IVA1 in 7 patients (13.2%), and IVA2 in 5 patients (9.4%); 12 cases (18.8%) showed large cell transformation.

**Fig. 1:**
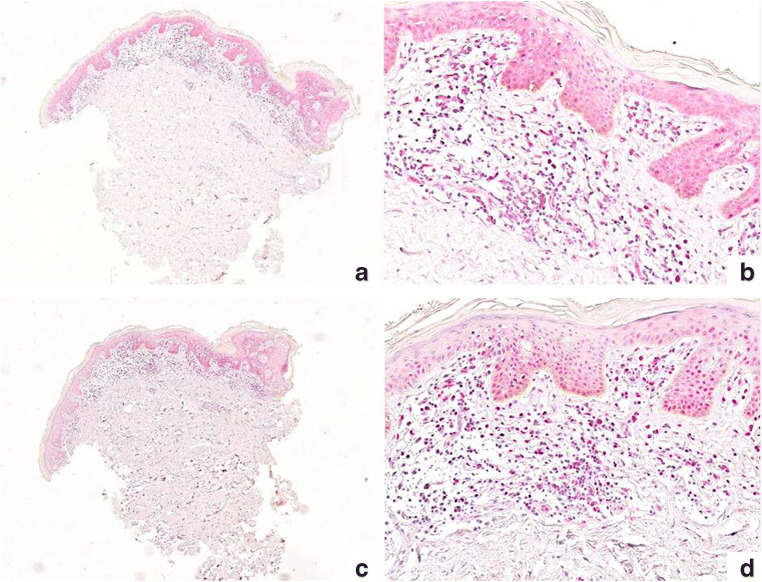
Immunohistochemical staining for CAF-1/p60 (**a** × 40; **b** × 200) and PARP-1 (**c** × 40; **d** × 200) in a case of patch stage MF. CAF-1/p60 resulted expressed only in 5% of the neoplastic cells while PARP-1 resulted overexpressed in 30% of neoplastic cells (both in epidermotropic that dermal lymphocytes)

**Fig. 2:**
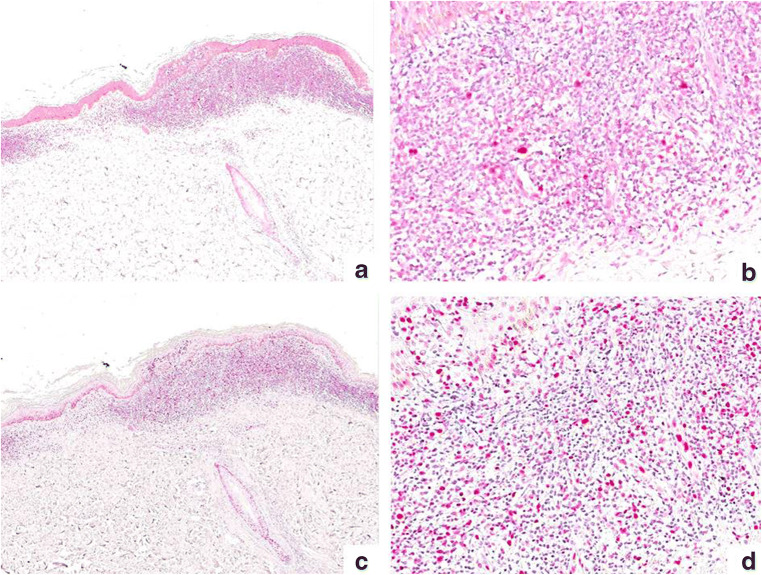
Immunohistochemical staining for CAF-1/p60 (**a** × 40; **b** × 200) and PARP-1 (**c** × 40; **d** × 200) in a case of plaque stage MF. CAF-1/p60 resulted overexpressed in 30% of the neoplastic cells while PARP-1 resulted overexpressed in 40% of neoplastic cells (mainly dermal lymphocytes)

**Fig. 3:**
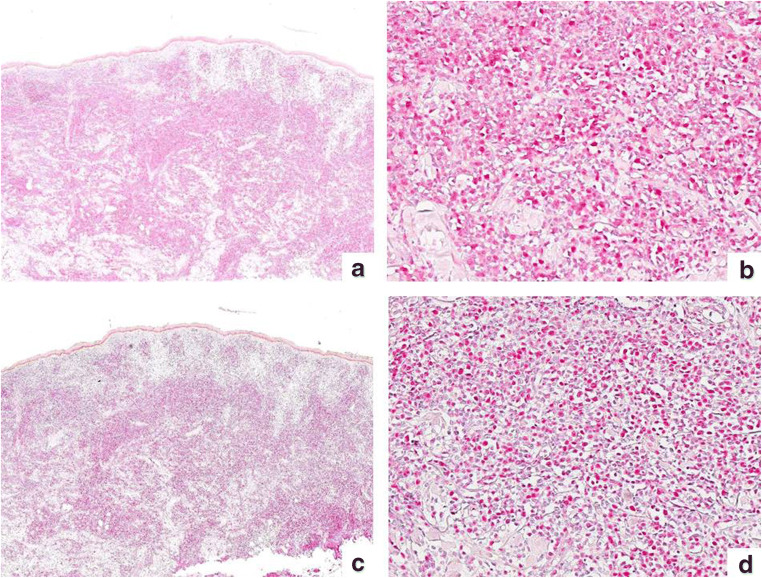
Immunohistochemical staining for CAF-1/p60 (**a** × 40; **b** × 200) and PARP-1 (**c** × 40; **d** × 200) in a case of tumor stage MF. CAF-1/p60 resulted overexpressed in 70% of the neoplastic cells and PARP-1 resulted overexpressed in 80% of neoplastic cells (dermal lymphocytes)

PARP-1 resulted overexpressed in 33 cases (57.9%), while CAF-1/p60 was overexpressed in 15 cases (26.8%) (Figs. 1, 2, and 3). Among the 12 cases with large cell transformation, PARP-1 and CAF-1/p60 immunostaining was successful in 11/12 cases, out of which 10 (90.9%) showed PARP-1 overexpression and 4 (36.4%) showed CAF-1/p60 overexpression; remarkably, considering only the large cells, both PARP-1 and CAF-1/p-60 were overexpressed in all 11 cases (100%).

A significant association was found between PARP-1 overexpression and CAF-1/p60 overexpression (*p* = 0.0025). PARP-1 was significantly associated with a MF stage > II (*p* = 0.034), while CAF-1/p60 was not (*p* = 1).

Follow-up data were available for 63 patients (98.4%); the mean follow-up duration was 52.9 months (range 12–180 months). At the last follow-up, 8 patients were dead (12.7%), 28 were alive with disease (44.4%), 15 were alive with complete remission (23.8%), and 6 were alive with unknown status of disease (19%).

**Fig. 4:**
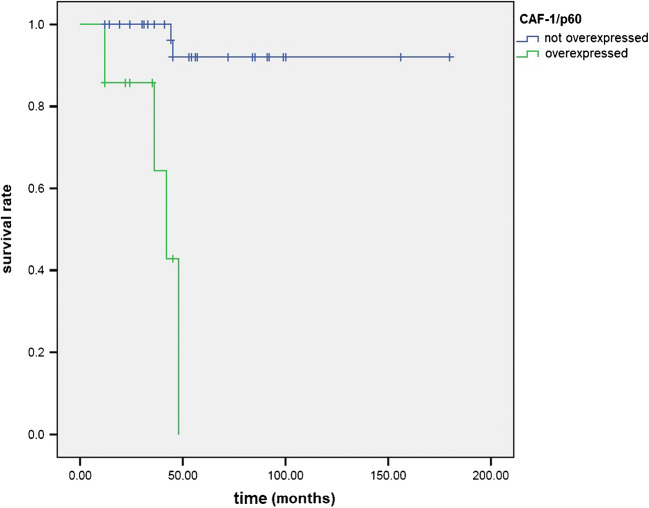
Kaplan-Meier curves for overall survival rate in patients with mycosis fungoides according to CAF-1/p60 expression (overexpressed vs not overexpressed)

CAF-1/p60 overexpression was significantly associated with shorter mean OS than CAF-1/p60 not overexpression (39 ± 4.066 vs 169.125 ± 7.386 months; *χ*^2^ = 21.729; *p* < 0.001) (Fig. 4); the prognostic significance of CAF-1/p60 overexpression was stronger than that of a MF stage ≥ II (139.586 ± 15.656 vs 141.6 ± 9.66 months; *χ*^2^ = 0.097; *p* = 0.756), ≥ IIB (93.645 ± 11.006 vs 167.556 ± 8.467 months; *χ*^2^ = 1.364; *p* = 0.243), and ≥ III (85.486 ± 15.681 vs 158.422 ± 10.33 months; *χ*^2^ = 1.624; *p* = 0.203) (Supplementary Figs. 1-3). On the other hand, PARP-1 overexpression was not associated with a significant decrease in the OS (89.123 ± 7.971 vs 163.972 ± 10.674 months; *χ*^2^ = 1.398; *p* < 0.237) (Supplementary Fig. 4); a simultaneous overexpression of PARP-1 and CAF-1/p60 was significantly associated with decreased OS (40.333 ± 4.507 vs 165.095 ± 8.149 months; *χ*^2^ = 14.916; *p* < 0.001) (Supplementary Fig. 5), although the association was weaker compared to CAF-1/p60 as stand-alone.

## Discussion

This study showed that, in MF, PARP-1 overexpression was associated with a more advanced MF stage, while CAF-1/p60 overexpression was associated with shorter OS.

The association between PARP-1 overexpression and CAF-1/p60 overexpression was significant but did not improve the prognostic stratification.

PARP-1 is a nuclear chromatin-associated protein involved in several biological processes including cell proliferation, apoptosis, malignant transformation, transcriptional regulation, and DNA repair. It is essential to the base excision repair of DNA single-strand breaks (SSBs). In response to DNA damage, PARP-1 senses and binds to DNA nicks and breaks, resulting in activation of catalytic activity, causing poly ADP-ribosylation of PARP-1 itself, as well as other acceptor proteins such as histones and topoisomerases. This modification stimulates the recruitment and activity of other components of DNA repair pathways [26]. In its absence, DNA SSBs accumulate and degenerate to DNA double-strand breaks (DSBs), which are not appropriately repaired if the BRCA pathway is deficient or dysfunctional. This is thought to explain the exquisite sensitivity to PARP inhibitors of tumors with BRCA inactivation, a concept called “synthetic lethality” [27, 28]. In addition to well-known activity in DNA strand breaks repair, PARP-1 has a role in other cellular pathways like DNA replication, chromatin structure modification, and cell cycle control. In particular, PARP-1 seems to have a role in stabilization and restart of the arrested replication fork and can facilitate nucleosome disassembly by histone PARylation resulting in chromatin relaxation facilitating DNA repair through the action of large protein complexes, such as the molecular chaperone chromatin assembly factor-1 (CAF-1) [27, 29–31]. CAF-1 is a histone chaperone conserved from yeast to human cells involved in the deposition of newly synthesized H3-H4 tetramers. CAF-1 interacts with proliferating cell nuclear antigen (PCNA), a DNA polymerase clamp for DNA polymerases, and co-localizes with PCNA at replication foci in the early S phase of the cell cycle, consistent with the idea that CAF-1 is recruited to DNA replication forks through its interaction with PCNA for nucleosome assembly [32].

PARP-1 and CAF-1/p60 nuclear proteins crosstalk with several molecular pathways involved in histone acetylation. The poly ADP-ribosylation of histones effectively functions like acetylation, maintaining chromatin nucleosomes in a fully relaxed, transcriptionally active state. Either PARP-1 [50] or CAF-1/p60 [43] overexpression has been reported in multiple types of cancer, and this has been related to histopathological grade and/or adverse clinical behavior. Their expression could help tumor cells to withstand genotoxic stress, by increasing their resistance to DNA-damaging agents and may result in radio-resistance and chemo-resistance [43, 50].

In this study, we found no prognostic value for PARP-1 expression in MF, as it was not associated with OS. This result does not confirm that reported in the previously published study by Lemchak et al., which showed an association between PARP-1 overexpression and MF progression. However, that study evaluating only 19 patients with MF represented a very preliminary assessment. Interestingly, Lemchak et al. showed that PARP inhibitors had a cytotoxic effect on Sezary cells, suggesting a possible use of PARP inhibitors in MF [25].

The results of our study do not contrast with such a possibility. In fact, we found that PARP-1 overexpression was significantly associated with a MF stage > II, providing a rationale for the use of PARP-1 inhibitors in advanced stages of MF.

Unlike PARP-1, we did not find an association between the MF stage and CAF-1/p60. Our result showed that CAF-1/p60 expression was a significant prognostic factor for the OS of patients with MF. CAF-1/p60 overexpression was indeed associated with a shorter OS more strongly than the MF stage. Remarkably, this result is consistent with previous studies focused on breast, oral, prostate, and salivary gland carcinomas, as well as in skin melanoma [44–48], strengthening the possible role of CAF-1/p60 as a “multi-tumoral” prognostic marker. These findings, although preliminary, may be a rationale for the development of targeted therapies against CAF-1/p60.

We previously demonstrated that a simultaneous overexpression of PARP-1 and CAF-1/p60 was a still more reliable prognosticator of poor outcome compared to CAF-1/p60 alone in oral carcinoma [51]. This finding was not confirmed in MF. Although a significant association between the overexpression of the two proteins was found, the simultaneous overexpression of PARP-1 and CAF-1/p60 appeared less reliable than CAF-1/p60 alone in predicting the risk of death in MF. However, given the association of PARP-1 and CAF-1/p60 with advanced stages and poor prognosis, respectively, it is possible to hypothesize that a combination of PARP inhibitors and anti-CAF-1/p60 targeted therapy might be effective in MF. In this regard, we previously showed that the inhibition of CAF-1/p60 made cancer cells more susceptible to PARP inhibitors [51].

Given the low number of patients assessed, our results should be considered preliminary. In fact, there were only few patients for each stage (e.g., only one patient was at stage III), making a correlation between protein expression and stage difficult. We believe our results are encouraging for further and larger studies in this field.

## Conclusion

In MF, PARP-1 overexpression is associated with advanced stage, while CAF-1/p60 is associated with decreased OS, resulting potentially useful as a prognostic marker. On this account, it is reasonable to hypothesize that PARP-1 inhibitors might be effective in advanced MF and that a targeted therapy against CAF-1/p60 might have a role as well. Further studies are encouraged in this regard.

## Electronic supplementary material

Supplementary Figure 1 Kaplan-Meier curves for overall survival rate in patients with mycosis fungoides according to tumor stage (I vs II-IV). (PNG 20 kb)

Supplementary Figure 2 Kaplan-Meier curves for overall survival rate in patients with mycosis fungoides according to tumor stage (I-IIA vs IIB-IV). (PNG 20 kb)

Supplementary Figure 3 Kaplan-Meier curves for overall survival rate in patients with mycosis fungoides according to tumor stage (I-II vs III-IV). (PNG 20 kb)

Supplementary Figure 4 Kaplan-Meier curves for overall survival rate in patients with mycosis fungoides according to PARP-1 expression (overexpressed vs not overexpressed). (PNG 22 kb)

Supplementary Figure 5 Kaplan-Meier curves for overall survival rate in patients with mycosis fungoides according to PARP-1 and CAF-1/p60 expression (both overexpressed vs at least one not overexpressed). (PNG 24 kb)

## Abbreviations

MF: Mycosis fungoides
PARP-1: Poly [ADP-ribose] polymerase 1
CAF-1: Chromatin assembly factor-1
CAF-1/p60: p60 subunit of CAF-1
