# Supplementary figures and images for: Role of chromatin assembly factor-1/p60 and poly [ADP-ribose] polymerase 1 in mycosis fungoides
Source: Virchows Arch. 2020 Oct 24;478(5):961–8. doi: 10.1007/s00428-020-02952-z (PMC8099834; doi:10.1007/s00428-020-02952-z)

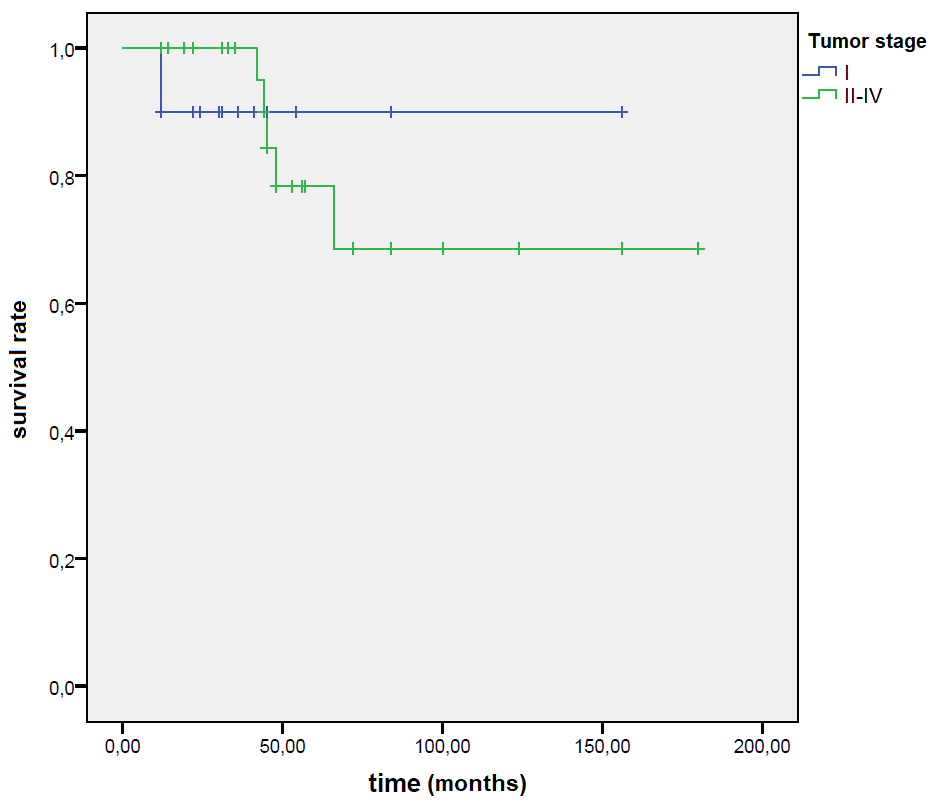

Supplement: Supplementary file 1 — Kaplan-Meier curves for overall survival rate in patients with mycosis fungoides according to tumor stage (I vs II-IV). (PNG 20 kb) [file 428_2020_2952_MOESM1_ESM.png]

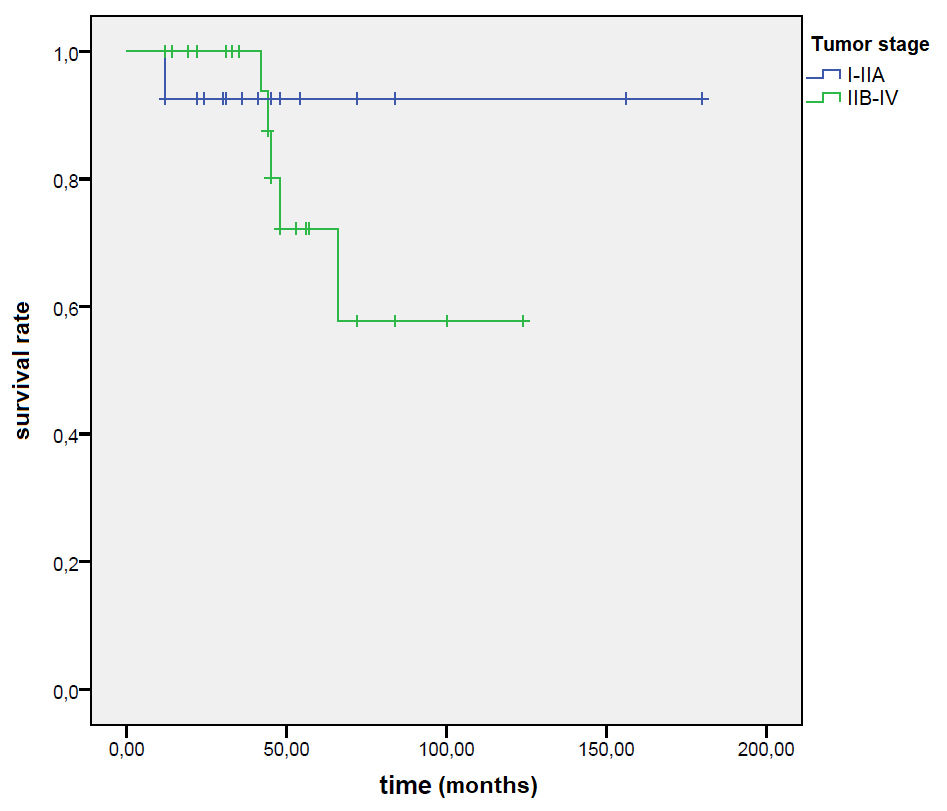

Supplement: Supplementary file 2 — Kaplan-Meier curves for overall survival rate in patients with mycosis fungoides according to tumor stage (I-IIA vs IIB-IV). (PNG 20 kb) [file 428_2020_2952_MOESM2_ESM.png]

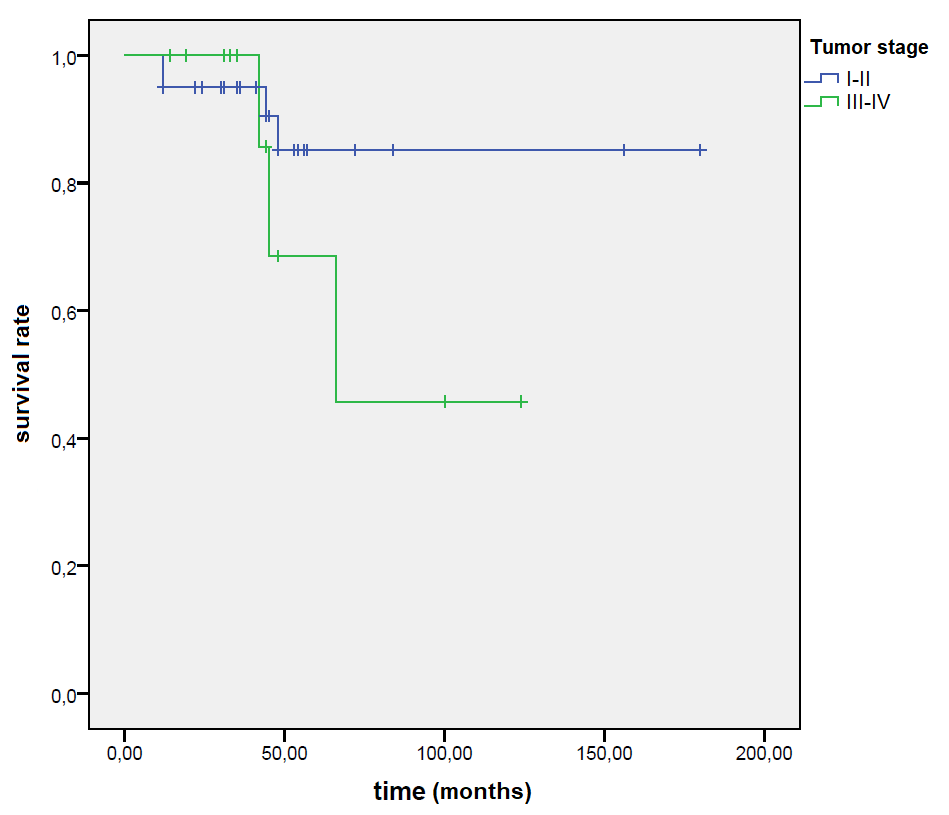

Supplement: Supplementary file 3 — Kaplan-Meier curves for overall survival rate in patients with mycosis fungoides according to tumor stage (I-II vs III-IV). (PNG 20 kb) [file 428_2020_2952_MOESM3_ESM.png]

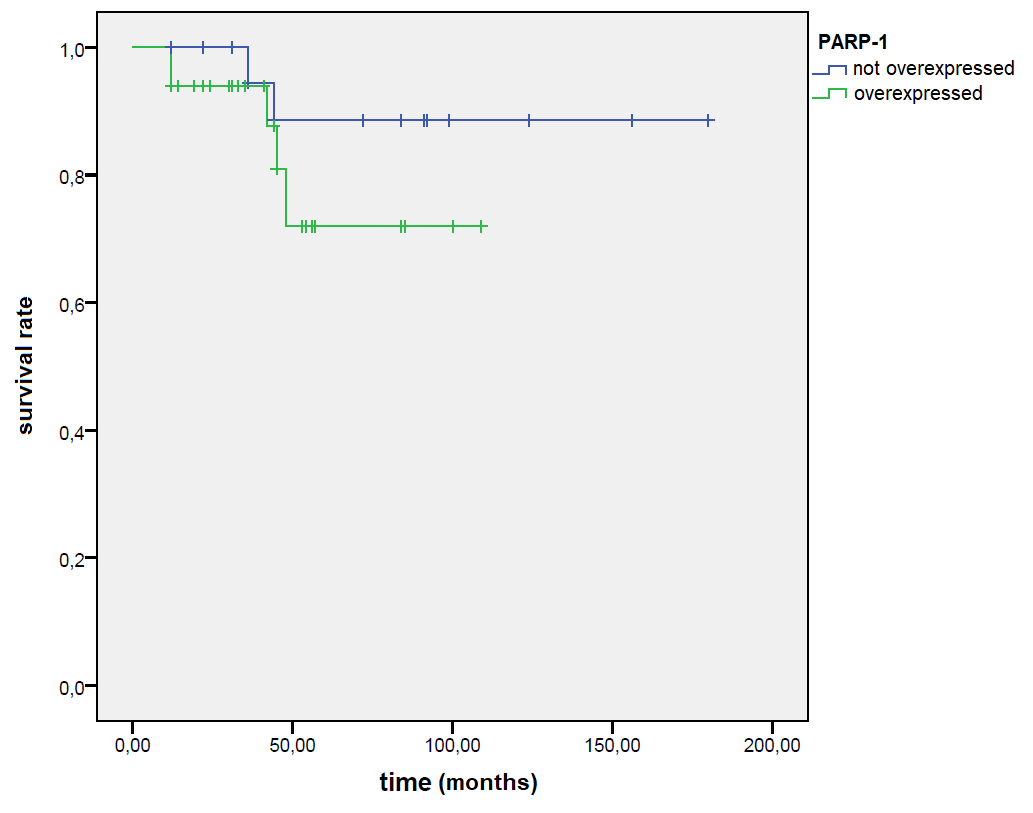

Supplement: Supplementary file 4 — Kaplan-Meier curves for overall survival rate in patients with mycosis fungoides according to PARP-1 expression (overexpressed vs not overexpressed). (PNG 22 kb) [file 428_2020_2952_MOESM4_ESM.png]

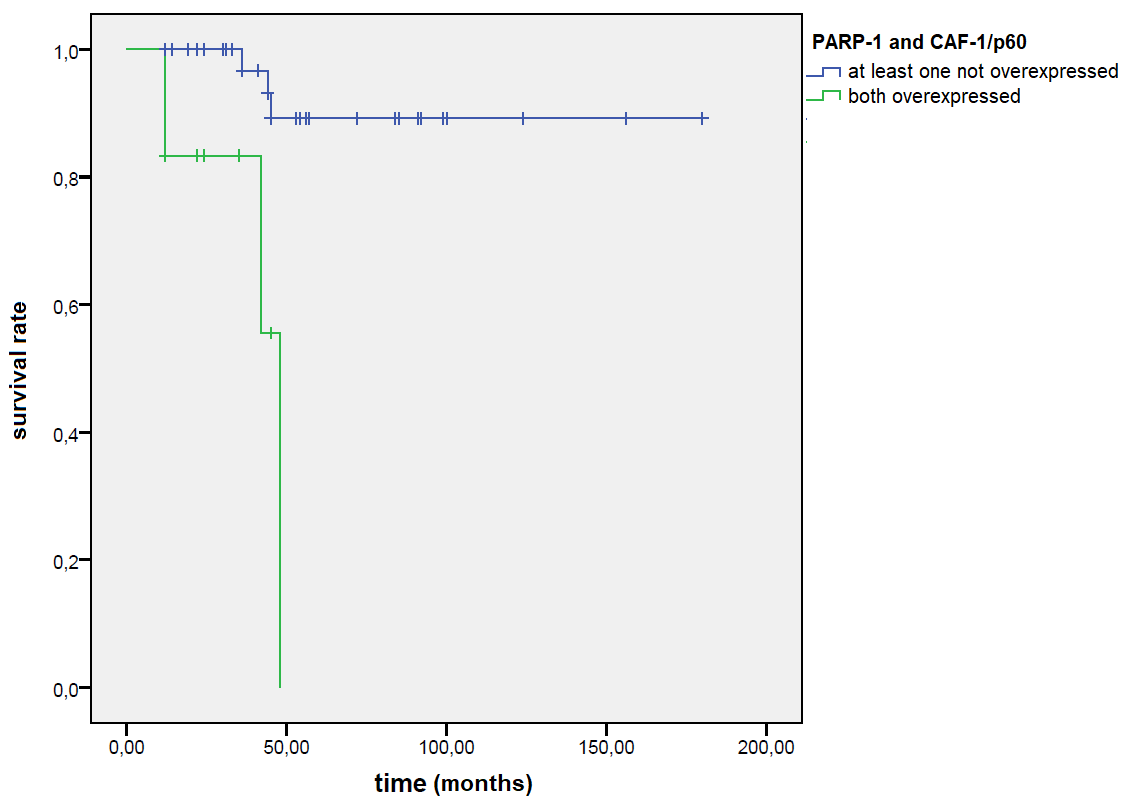

Supplement: Supplementary file 5 — Kaplan-Meier curves for overall survival rate in patients with mycosis fungoides according to PARP-1 and CAF-1/p60 expression (both overexpressed vs at least one not overexpressed). (PNG 24 kb) [file 428_2020_2952_MOESM5_ESM.png]
